# Neural Processing of Naturalistic Echolocation Signals in Bats

**DOI:** 10.3389/fncir.2022.899370

**Published:** 2022-05-18

**Authors:** M. Jerome Beetz, Julio C. Hechavarría

**Affiliations:** ^1^Zoology II, Biocenter, University of Würzburg, Würzburg, Germany; ^2^Institute of Cell Biology and Neuroscience, Goethe University Frankfurt, Frankfurt, Germany

**Keywords:** biosonar, neural coding, naturalistic stimuli, bats, acoustic stream, neuroethology

## Abstract

Echolocation behavior, a navigation strategy based on acoustic signals, allows scientists to explore neural processing of behaviorally relevant stimuli. For the purpose of orientation, bats broadcast echolocation calls and extract spatial information from the echoes. Because bats control call emission and thus the availability of spatial information, the behavioral relevance of these signals is undiscussable. While most neurophysiological studies, conducted in the past, used synthesized acoustic stimuli that mimic portions of the echolocation signals, recent progress has been made to understand how naturalistic echolocation signals are encoded in the bat brain. Here, we review how does stimulus history affect neural processing, how spatial information from multiple objects and how echolocation signals embedded in a naturalistic, noisy environment are processed in the bat brain. We end our review by discussing the huge potential that state-of-the-art recording techniques provide to gain a more complete picture on the neuroethology of echolocation behavior.

## Introduction

Bats acoustically orient by broadcasting echolocation sequences and extracting spatial information from echoes, a strategy referred to as echolocation behavior. The accessibility of naturalistic orientation signals makes the investigation of the neural underpinnings of echolocation behavior a promising field of research. Traditionally, electrophysiological studies are conducted by using synthesized echolocation signals that represent only portions of naturalistic echolocation signals (Dear and Suga, 1995; Kössl et al., 2015; Macías et al., 2020b). Hereby, both the call dynamics and the temporal context of acoustic signals, i.e., the organization of echolocation signals in sequences, has often been neglected. This is surprising when considering the substantial effects of stimulus rate on neural processing (O’Neill and Suga, 1982; Wong et al., 1992; Wu and Jen, 1996; Galazyuk et al., 2000; Smalling et al., 2001; Zhou and Jen, 2006; Macías et al., 2022). One possible reason why many scientists use synthesized acoustic stimuli instead of naturalistic echolocation signals is, besides from the fact that artificial stimuli are well controllable, the challenge of recording the echolocation signals immediately before they arrive at the bat’s ears. To get along with that challenge, it is desirable to place a microphone close to the bats’ ears (Hase et al., 2022), a complicated task when considering that commercially available recording devices are usually too heavy to be carried by bats. Therefore, some scientists adopted a pendulum paradigm that allows to place a bat together with acoustic recording devices in mass of a pendulum (Henson et al., 1982; Kobler et al., 1985; Gaioni et al., 1990; Fitzpatrick et al., 1991; Vater et al., 2003; Macías et al., 2016b; Beetz et al., 2021). During the forward swing, the bat broadcasts echolocation calls that are recorded together with echoes by an ultrasound sensitive microphone. These acoustic signals can later be used as naturalistic echolocation signals presented to passively listening bats (Beetz et al., 2016a,b, 2017, 2018). Another approach, but leading to comparable acoustic recordings, is to train bats resting on a perch while mechanically approaching a target that the bat must ensonify (Macías et al., 2018). However, by far the most naturalistic and desirable approach is to record neural signals directly from echolocating bats (Kothari et al., 2018).

Before focusing on recent neurophysiological findings, the review gives a brief historical background on the discovery of echolocation behavior, and an overview on how echolocation information conveys spatial information. This is of particular importance when evaluating the stimulus design used for neural recordings. Later, the review focuses on four aspects that scientists should consider when investigating neural coding of naturalistic echolocation signals: (i) temporal context and dynamics of the stimulus; (ii) complex echolocation scenes composed of multiple objects or multi-reflective substrates; (iii) echolocation signals embedded in a naturalistic acoustic context; (iv) neural recordings from actively echolocating and flying bats.

## Brief History of Bat Echolocation

Observations on bat navigation date to the 18th century when Lazzaro Spallanzani described that bats with occluded eyes perform rapid flight maneuvers without colliding with surrounding obstacles (Dijkgraaf, 1960; Griffin, 2001). Subsequent experiments by Louis Jurine of Geneva demonstrate that bats use their auditory system for navigation because when the bats’ ear canals were plugged, their navigational capabilities drastically dropped, and they collided with obstacles. Hahn (1908) conducted similar experiments and came to the same conclusions. Because scientists could not detect any sounds that the bats may use for orientation, it remained mysterious how the auditory system helps the bats to orient in darkness. Hartridge (1920) proposed that bats emit high frequency calls, inaudible to humans and that they extract spatial information from the echoes. Ironically, this hypothesis could not be tested until G. W. Pierce invented an ultrasound detector about two decades later and confirmed Hartridge’s hypothesis together with Donald R. Griffin (Pierce and Griffin, 1938; Griffin, 1944, 1958). High frequency signals are spatially directed and their short wavelengths allow reflections off small targets, optimal to hunt for tiny insects (Boonman et al., 2013). The shortcoming of high frequency calls is that they rapidly attenuate with distance limiting the working range of echolocation to just a few meters (Grinnell and Griffin, 1958; Kick, 1982; Lawrence and Simmons, 1982b). To navigate over long distances, bats often use a combination of auditory and visual information (Williams and Williams, 1967, 1970; Boonman et al., 2013). In addition, a magnetic compass is discussed that assists bats to find their way over long-distance migrations (Holland et al., 2006, 2010; Wang et al., 2007b).

## Acoustic Parameters of Echolocation Signals

**FIGURE 1:**
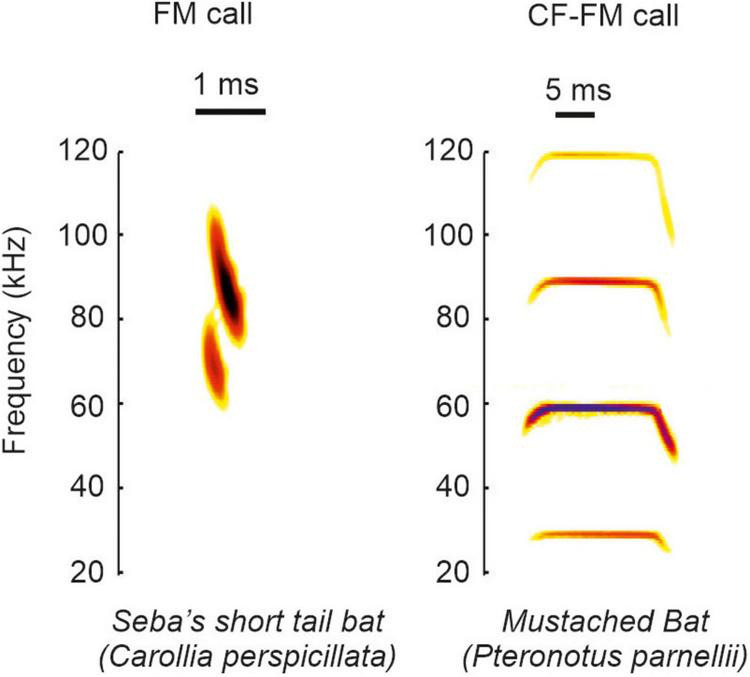
Example calls of FM and CF-FM bats. The example spectrograms shown correspond to vocalization from the species *Carollia perspicillata* and *Pteronotus parnellii*.

Based on the call structure, bats can be classified into species emitting frequency modulated calls (FM-bats) or a combination of constant frequency and frequency modulated call-components (CF-FM bats) (Neuweiler, 1990; Altringham, 2011; Figure 1). The FM covers a broad frequency range (Simmons and Young, 2010) and it is suited to analyze the object’s position, structure, and shape (Simmons, 1973; Simmons et al., 1979; Simmons and Stein, 1980; Surlykke et al., 1993; Faure and Barclay, 1994; Jensen and Miller, 1999; Siemers and Schnitzler, 2004). The narrowband CF component lasts longer than the FM and some insectivorous bats use it to detect flying insects that cause amplitude and frequency modulations in the echoes (Neuweiler, 1990). The ecological diversity of bats is reflected in the acoustic call properties. Each bat species adopts different calls, and each individual can dynamically change different call portions (for review see Griffin and Novick (1955), Neuweiler (1989, 1990), Jones (1999), Schnitzler and Kalko (2001), Brinklov et al. (2010)). The dynamics in call design imply that there does not exist a unique call template for each species. Because each call may slightly differ from preceding calls, it is challenging to discuss the behavioral relevance from neurophysiological findings obtained with a single call template used as acoustic stimulus.

Not only the call variability may affect neural processing but also the sequence character of the emitted signals. Each echo represents an acoustic snapshot of the surrounding, comparable to a single frame of a movie. As with adding single frames at a certain rate to create movie scenes, the sequence character of emitted echolocation calls results in a smooth and quasi-continuous representation of the surroundings. By dynamically adjusting the call rate, bats control the spatial accuracy of their echolocation system. High spatial accuracy is important when bats navigate in highly cluttered or unfamiliar environments or when zooming into objects or prey (Kick, 1982; Roverud and Grinnell, 1985a; Surlykke and Moss, 2000; Petrites et al., 2009; Hiryu et al., 2010; Barchi et al., 2013; Falk et al., 2014; Kothari et al., 2014; Sändig et al., 2014; Knowles et al., 2015; Wheeler et al., 2016; Beetz et al., 2019). Immediately before the catch, some bats reach call rates higher than 200 Hz (Griffin, 1953; Schnitzler et al., 1987; Jones and Rayner, 1988; Kalko and Schnitzler, 1989; Kalko, 1995). Taken together, the dynamics in call design and call rate may affect neural processing and must be carefully considered when investigating how naturalistic echolocation signals are processed in the bat brain.

## What Spatial Information Is Conveyed by Echoes?

**FIGURE 2:**
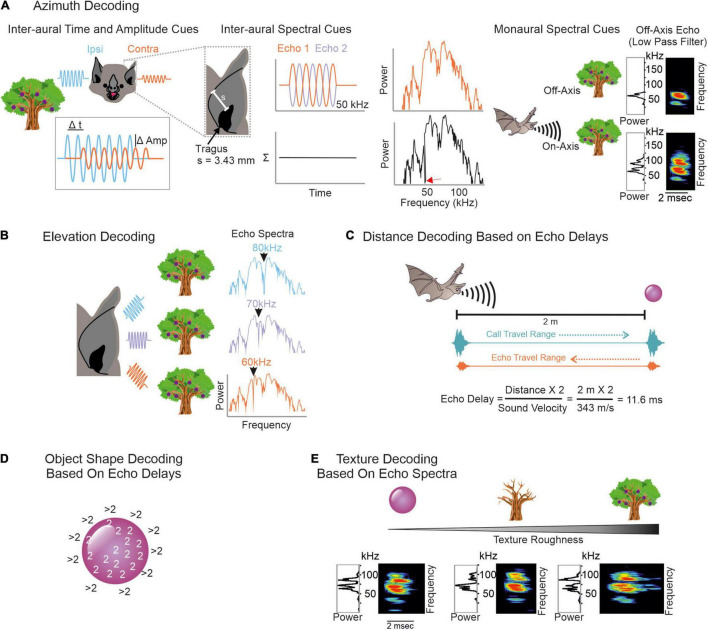
Overview on decoding spatial information from echoes. **(A)** To infer an object’s azimuth, bats can use inter-aural amplitude, time (*left*), and spectral cues (*middle*), but also monaural spectral cues (*right*). Ipsilateral echoes (*blue waves*) are higher in amplitude (*Amp*) and reach the ipsilateral ear earlier than the contralateral ear (*orange waves*). Contralateral echoes bypass the bat’s head which creates spectral notches due to multiple reflections. To understand how spectral notches are generated, we assume that the echo gets reflected off the bat’s pinna. This evokes a second echo that temporally overlaps with the first echo. By assuming a pinna-tragus distance of 3.43 mm and considering that the second echo travels back to the tragus, sound waves with a wavelength of 6.86 mm (or 50 kHz) are phase shifted by 180° between both echoes. This creates a spectral notch at 50 kHz (*red arrow* in black power spectrum). Based on characteristic spectral notches, contralateral echoes are discriminable from ipsilateral ones. Because of the frequency dependent directionality, echoes from off-axis objects mainly contain low frequency portions. Echoes from on-axis objects additionally contain high frequency portions (Surlykke et al., 2009a; Simmons, 2012, 2014; Wohlgemuth et al., 2016b). **(B)** Elevation decoding is based on elevation-dependent spectral-notches in the echoes. **(C)** Target distance is computed based on echo-delays. Both call and echo travel the distance between the bat and the object at sound velocity. This leads to distance-dependent echo delays to which combination-sensitive neurons in the auditory system of bats are tuned to. **(D)** To determine the object’s shape, the edges can be scanned by computing echo-delays. As soon as the calls get reflected off objects in the background, the echo delays abruptly increase. **(E)** Texture roughness is determined by spectral cues like spectral notches.

To conceptualize how echoes convey spatial information, we imagine a simplified scenario, where a frugivorous FM-bat detects a fruit tree. First, the bat localizes the tree relative to its current location. It computes the tree position along the horizontal axis (azimuth) by using binaural cues (Simmons et al., 1983; Obrist et al., 1993; Fuzessery, 1996; Firzlaff and Schuller, 2003). Echoes arrive earlier and with a higher intensity to the ipsilateral ear than to the contralateral ear (Figure 2A, *left column*). Because of the bat’s small head size, inter-aural time differences may be too short and thus less relevant to decode the tree’s azimuth position (Grothe and Park, 1998). With their fine frequency tuning, bats can also use inter-aural spectral differences to infer the tree’s azimuthal position. To reach the contralateral ear, the echo passes the bat’s head. This creates multiple echoes (Wotton et al., 1995; Aytekin et al., 2004) that partially overlap and thus creates spectral notches in the echo reaching the contralateral ear (Figure 2A, *middle column, see also Figure legend for details*). Spectral differences of echoes reaching the ipsi- and contralateral ear can be used to determine the azimuthal position of the echo source (Fuzessery, 1996; Aytekin et al., 2004). In addition to inter-aural parameters, bats can also use monaural spectral cues, i.e., the energy content at particular frequency bands to infer the object’s azimuth (Bates et al., 2011; Wohlgemuth et al., 2016b; Figure 2A, *right column*).

To adjust the flight height and avoid collisions, the bat must know the tree’s height. Due to the characteristic anatomy of the outer ear, echoes get reflected off ear parts in an elevation-dependent manner (Grinnell and Grinnell, 1965; Lawrence and Simmons, 1982a; Wotton et al., 1995; Firzlaff and Schuller, 2003; Aytekin et al., 2004; Chiu and Moss, 2007; Hoffmann et al., 2015). The position and number of spectral notches depend on the object’s elevation (Fuzessery, 1996; Aytekin et al., 2004). With increasing elevation, the notch frequency increases (Wotton et al., 1995; Firzlaff and Schuller, 2003; Aytekin et al., 2004; Wohlgemuth et al., 2016b; Figure 2B). Behavioral studies in the FM-bat *Eptesicus fuscus* showed that they indeed use spectral notches for elevation processing (Wotton et al., 1996; Wotton and Simmons, 2000).

For distance processing, bats measure the delay between call emission and echo arrival, also referred as “echo delay” (Figure 2C) (Hartridge, 1945; Nordmark, 1960; Cahlander et al., 1964; Simmons, 1973, 1989). Because acoustic signals travel at an approximate constant speed, the echo delay increases with distance, 1 ms per 17 cm. Distance processing based on echo-delays only works until there is a detectable delay between call and echo. If the object is just a few centimeters away from the bat, call and echo temporally overlap and bats use spectral information, including notches arising from call-echo interferences to process the object’s distance (Pye, 1960, 1961a,b). Neurons sensitive to spectral notches have been well described in the inferior colliculus and auditory cortex of the insectivorous FM-bat *Eptesicus fuscus* (Sanderson and Simmons, 2000, 2002). Along the same line of argument, if two objects are very close to each other, e.g., a fruit in front of clutter, then both objects create temporally overlapping echoes. Some neurons of the inferior colliculus of *E. fuscus* respond differently to objects associated with clutter than in the absence of clutter allowing a reliable sonar object discrimination in a cluttered environment (Allen et al., 2021).

Bats also determine the object’s shape and texture with echo information. Here, they profit again from different strategies. One possibility is to scan object edges (Yovel et al., 2010) by using echo delay information (Figure 2D). Many bats living in cluttered environments often hover in front of objects (Griffin and Novick, 1955; Neuweiler, 1989, 1990; Schmidt et al., 2000; von Helversen and von Helversen, 2003). By keeping a constant object-distance and carefully pinpointing the sonar beam along object edges, bats can compute the object’s shape. As soon as the sonar beam points off the object, the echolocation call hits an object in the background and the echo delay dramatically shifts to long delays. Note that while these ideas have been discussed extensively and they have strong theoretical support, whether the bats use echo delay information to infer the object’s shape remains unclear. To date, most neural data have been obtained in head-restrained bats that were under anesthesia. To understand the purpose of hovering and especially its neuronal control, future studies should follow a more naturalistic approach by recording from vocalizing bats hovering in front of objects.

Objects spatially tightly embedded in highly cluttered backgrounds might result in delay differences of less than a millisecond, making object shape determination based on echo delay information challenging (Simmons et al., 1974; Simmons and Chen, 1989). Under these conditions, it might be easier for the bat to infer object shapes and textures with spectral information (Simmons et al., 1974, 1990, 1998; Simmons, 1989; Mogdans et al., 1993; Simon et al., 2006; Figure 2E). Neurophysiological studies demonstrate that subcortical and cortical neurons, indeed selectively respond to spectral notches (Sanderson and Simmons, 2002; Firzlaff et al., 2006; Macías et al., 2020a).

## Influence of the Temporal Context on Neural Processing

**FIGURE 3:**
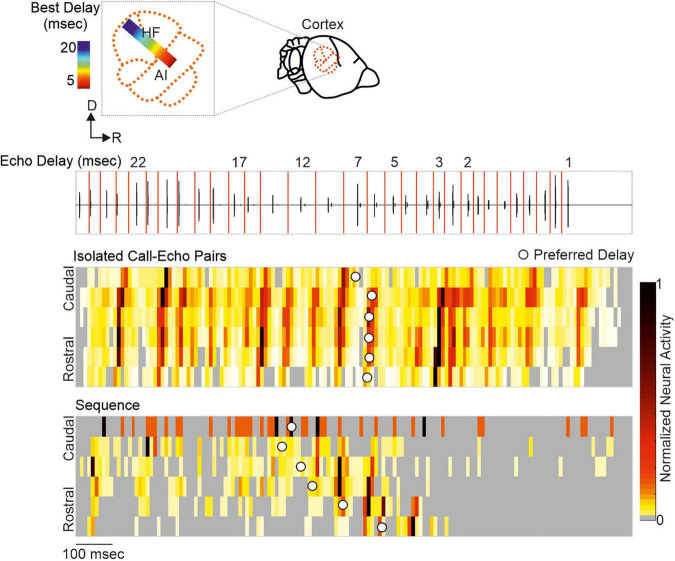
A neural activity wave travels across the cortex when the bat is stimulated with a naturalistic echolocation sequence of an approach flight, but it is absent when presenting the same call-echo pairs in randomized order. Top: Schema of the chronotopic gradient of the auditory cortex in *Carollia perspicillata*. Neurons tuned to long delays (∼ 20 ms) are clustered in caudal regions while short delays (∼ 2 ms) are primarily processed in rostral regions of the cortex. Bottom: Oscillogram of the stimulus. Red vertical lines border the call-echo pairs. Heatmaps represent the neural activity of six simultaneously recorded units in response to temporally isolated call-echo pairs **(Top)** and to the naturalistic echolocation sequence **(Bottom)**. The naturalistic echolocation sequence contained the same acoustic information as the call-echo pairs that were played randomly with a 500 ms inter-stimulus interval. Only the temporal context, i.e., stimulus history and the stimulus rate differ between both stimulation protocols. Units are ordered along the rostro-caudal axis. Preferred echo delays, median time points of the neural responses of each unit are indicated as white dots. Data adapted from Beetz et al. (2016b).

Neurons encoding object distances belong to one of the best described neuron types in bat research (Feng et al., 1978; O’Neill and Suga, 1979; Sullivan, 1982b; Fitzpatrick et al., 1993; Portfors and Wenstrup, 1999; Hagemann et al., 2011; Wenstrup and Portfors, 2011; Hechavarría and Kössl, 2014; Macías et al., 2016a). While being diffusely organized in the inferior colliculus, delay-tuned neurons of the auditory cortex are, in many bat species, topographically organized in a chronotopic map (for review see Kössl et al. (2014)). Neurons tuned to long echo-delays and thus encoding long target-distances are clustered in different cortical areas than neurons tuned to short echo-delays. When a bat approaches a target during flight, the echo delays progressively shorten (*Oscillogram* in Figure 3). By considering the chronotopic organization, it was discussed that an activity wave moves along a target-distance gradient in the cortex when the bat approaches a target (Hechavarría et al., 2013; Bartenstein et al., 2014; Kössl et al., 2014). This activity wave was experimentally confirmed by placing multi-electrode arrays along the target-distance gradient of an anesthetized frugivorous bat *Carollia perspicillata* and recording simultaneously from multiple neurons that were tuned to different echo delays (Beetz et al., 2016b). When the bats were stimulated with a naturalistic echolocation sequence of an approach flight, an activity wave moved from caudal to rostral cortex regions. Caudal neurons encoding long echo-delays respond stronger to the first half of the sequence while the neural activity propagated toward rostral neurons during the course of the sequence. Unexpectedly, this activity wave was absent when presenting the bats isolated call-echo pairs in a randomized order and with at least 500 ms of inter-stimulus interval (compare response to “Isolated Call-Echo Pairs” with response to “Sequence” in Figure 3). Although both stimulus protocols “Isolated Call-Echo Pairs” and “Sequence” contain the same echolocation information, they evoke totally different neural responses in the cortex. These different responses can be explained when considering the stimulus history and acoustic rates carried by the stimuli. The high acoustic rate represented in the naturalistic sequence evokes substantial neuronal suppression in the cortex. However, instead of completely silencing the cortex, the neurons partially recover from suppression at neuron-specific echo delays (Beetz et al., 2016b). Thus, cortical suppression in response to the echolocation sequence, renders an otherwise highly responsive cortex into a selective cortex that maps the distance information of an approach flight.

**FIGURE 4:**
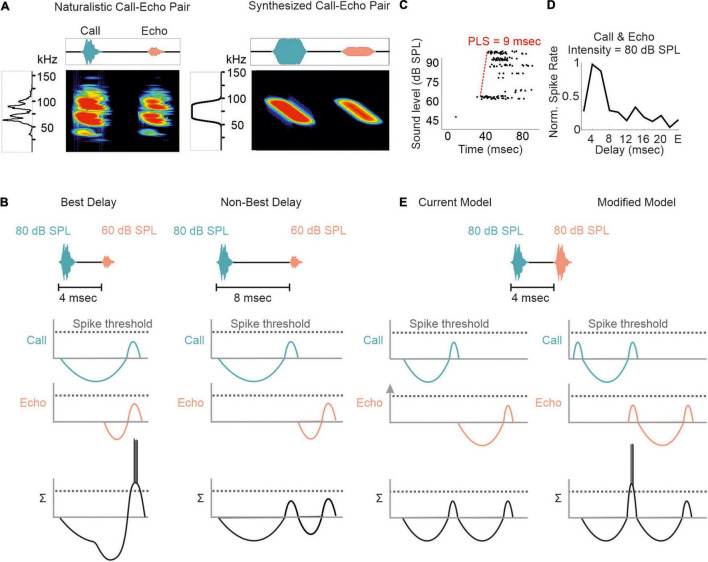
Proposed models of delay tuning in FM-bats. **(A)** Oscillogram, spectrogram, and power spectrum of a naturalistic call-echo pair of *C. perspicillata* (*left*) and a synthesized call-echo pair (*right*). For simplicity the power spectrum represents the call only. **(B)** Proposed model of delay tuning in FM-bats. Post-synaptic responses of a delay-tuned neuron are schematically shown in response to the neuron’s best delay (4 ms, *left*) and a non-best delay (8 ms, *right*). Post-synaptic inputs from call and echo are depicted in green and orange, respectively. The summed response (Σ) of the delay-tuned neuron is shown in black. Note that the neuron spikes only when the inhibitory rebounds from call and echo coincide. **(C)** Rate-level function of a cortical delay-tuned neuron showing a paradoxical latency shift (PLS) of about 9 ms. Each dot of the raster plot represents a spike **(D)** Delay-tuning curve from a cortical neuron of *C. perspicillata* that is sensitive to equally intense call and echo (80 dB SPL). **(E)** The current model of delay-tuning in FM-bats cannot explain delay tuning to equally intense call and echo (*left*). By adding an initial excitatory component, delay-tuning to equally intense call and echo can be explained (*right*). In the modified model, the post-inhibitory rebound from the call response coincides with the initial excitatory response to the echo, thus passing the spike threshold. Data shown in **(C)** are adapted from Hechavarría and Kössl (2014).

Although substantial effects of stimulus rate on tuning sharpness have been documented in former studies (O’Neill and Suga, 1982; Wong et al., 1992; Wu and Jen, 1996, 2006b; Galazyuk et al., 2000; Smalling et al., 2001; Jen et al., 2002; Zhou and Jen, 2006), delay tuning is commonly examined by using synthesized call-echo pairs as acoustic stimuli (Dear and Suga, 1995; Hagemann et al., 2010; Kössl et al., 2012, 2015; Hechavarría et al., 2013; Macías et al., 2016a, 2020b). These stimuli only mimic portions of the echolocation signals (Figure 4A) and are far beyond a naturalistic stimulus context (Sullivan, 1982b; Casseday and Covey, 1996; Galazyuk et al., 2005; Feng, 2011; Wenstrup et al., 2012; Hechavarría and Kössl, 2014; Suga, 2015). The current model on delay tuning in FM-bats was designed based on results obtained under artificial stimulus conditions. According to this model, each acoustic signal evokes an inhibition followed by a rebound excitation (Figure 4B). Hereby, the duration of inhibition depends on the signal amplitude. The higher the signal amplitude, the longer the inhibition and the longer the response latency, a phenomenon also referred to as paradoxical latency shift (PLS) which is widespread in delay-tuned neurons of FM-bats (Sullivan, 1982a,b; Berkowitz and Suga, 1989; Klug et al., 2000; Galazyuk and Feng, 2001; Galazyuk et al., 2005; Wang et al., 2007a; Ma and Suga, 2008; Feng, 2011; Hechavarría and Kössl, 2014). Since echolocation calls are more intense than echoes, delay-tuned neurons respond with longer latencies to calls than to echoes. If the amount of PLS is as long as the echo delay, then both subthreshold responses coincide and pass the spike threshold (*“Best Delay” column* in Figure 4B). Depending on the amount of PLS, different neurons have specific echo delays to which they respond best (Sullivan, 1982b; Berkowitz and Suga, 1989; Galazyuk et al., 2005; Feng, 2011; Hechavarría and Kössl, 2014). Despite the elegance of PLS, it does not fully explain the mechanisms underlying delay tuning in FM-bats. Some delay-tuned neurons show no PLS, and delay tuning can even be observed when call and echo are equally intense (Grinnell, 1963; Hechavarría and Kössl, 2014; Beetz et al., 2016b; Figures 4C,D). The latter cannot be explained with a PLS because excitatory rebounds from call and echo would never coincide (“*current model*” in Figure 4E). Based on results from extracellular recordings from lightly anesthetized and passively listening bats, we modified the current model of delay tuning. If a brief subthreshold excitation precedes the inhibition, ending up with two excitatory components (initial excitation and inhibitory rebound) and one inhibitory component evoked by each acoustic signal, then the inhibitory rebound in response to the call may coincide with the initial excitation in response to the echo (*“modified model”* in Figure 4E). This may explain delay tuning to equally intense acoustic signals and in absence of PLS. Unfortunately, no intracellular data from the auditory cortex of FM-bats exist to test the existence of a subthreshold initial excitation. So far, all intracellular recordings in FM-bats focused on the inferior colliculus (Covey et al., 1996; Xie et al., 2007; Peterson et al., 2008; Voytenko and Galazyuk, 2008; Li et al., 2010). Importantly, many neurons from the auditory midbrain show an initial excitation followed by inhibition (Suga, 1964; Covey et al., 1996), a process mediated a process mediated by GABA (Jen et al., 2002; Zhou and Jen, 2002; Wu and Jen, 2006a,b). However, it is noteworthy that changes in signal duration can substantially change the post-synaptic potentials of a collicular neuron (Covey et al., 1996). The high post-synaptic dynamics induced by slight stimulus changes raises concerns about the transferability of post-synaptic potentials measured with artificial stimuli. To what extent delay-tuned neurons of the cortex show initial excitatory responses followed by inhibition and post-inhibitory rebounds and whether these response components contribute to the neural mechanisms of delay tuning awaits to be answered.

**FIGURE 5:**
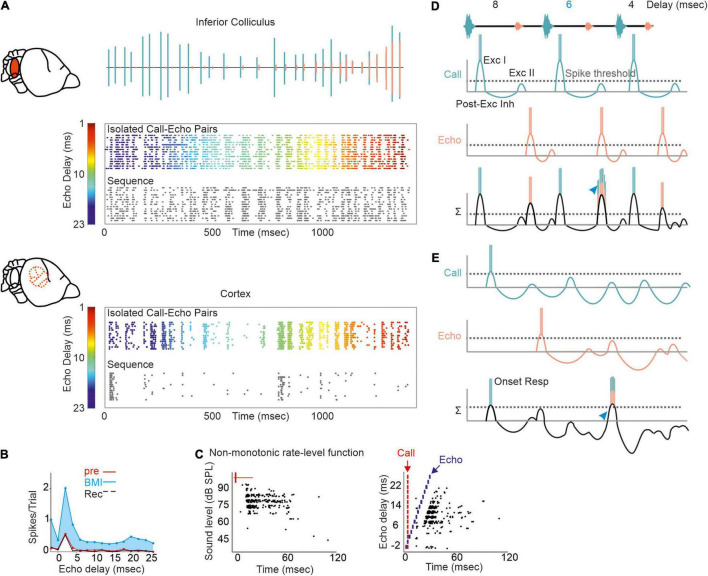
Proposed mechanism of processing naturalistic echolocation sequences. **(A)** Neural response to isolated call-echo pairs (colored raster plot) presented in randomized order and with 500 ms interstimulus time-interval and to a naturalistic echolocation sequence (black raster plot) from the inferior colliculus (*upper*) and auditory cortex (*lower*) of *C. perspicillata*. The naturalistic echolocation sequence is shown as oscillogram where green and orange events indicate calls and echoes, respectively. **(B)** Effect of GABA blocking in a cortical delay-tuned neuron. Blockage with bicuculline methiodide (BMI) reduces the spike rate while the delay-tuning was unaffected. **(C)** Rate-level function (*left*) and delay tuning (*right*) visualized as raster plots of one cortical neuron. Note that the neuron responds non-monotonically with more spikes to faint than to intense sound levels (*left*). Each black dot represents a spike. **(D,E)** Proposed models of processing echolocation sequences in the inferior colliculus **(D)** and auditory cortex **(E)**. For simplicity, the synaptic input to delay-tuned neurons is demonstrated for a sequence containing three call-echo pairs with echo-delays of 8, 6, and 4 ms. Synaptic inputs evoked by calls and echoes are indicated in green and orange. Both inputs are linearly summed (Σ) to explain the post-synaptic potential and spike output of the delay-tuned neurons. Although, responding with spikes to each acoustic event, the collicular neuron shows a faciliatory response [blue arrowhead in **(D)**] to the call-echo pair in which the echo follows the call by 6 ms. The cortical neuron responds more selectively to the 6 ms call-echo pair of the sequence (blue arrowhead). Respectively, data shown in **(A–C)** are adapted from Hechavarría and Kössl (2014) and Beetz et al. (2017).

What is even more underexplored is how the temporal dynamics of excitation and inhibition influence the neural responses to naturalistic echolocation sequences in FM bats. At the level of the auditory midbrain, neural suppression in response to the echolocation sequence improves the neural tracking ability of acoustic signals by increasing the signal-to-noise ratio of the neural response (Beetz et al., 2017). While many collicular neurons are adapted to encode the time points of acoustic signals in an echolocation sequence (Sanderson and Simmons, 2005; Beetz et al., 2017; Macías et al., 2018), forward suppression vastly deteriorates this tracking ability at the cortex level (Bartenstein et al., 2014; Beetz et al., 2016b; Figure 5A). In contrast to *C. perspicillata* and *Phyllostomus discolor*, cortical suppression is weaker in the FM-bat *Tadarida brasiliensis* and some neurons well represent the time points of acoustic signals (Macías et al., 2022). Hereby, the spike timing precision increases with the stimulus rate and thus improves the neurons’ tracking ability (Macías et al., 2022) similar to what has been found in the inferior colliculus of *C. perspicillata* (Beetz et al., 2017). In contrast to this, neural suppression in the inferior colliculus of *E. fuscus* is stronger than in *C. perspicillata*, resulting in some neurons selectively responding to particular call-echo pairs in a naturalistic echolocation sequence (Macías et al., 2018). Altogether, the degree and site of neural suppression can differ across bat species, an interesting comparative effect on processing echolocation sequences.

Regional differences in GABA-mediated inhibitions in the inferior colliculus and auditory cortex may explain the different suppression strength seen in the midbrain and cortex of *C. perspicillata*. While blocking GABA abolishes PLS and affects delay tuning in the inferior colliculus (Galazyuk and Feng, 2001; Feng, 2011), at cortical level, PLS and delay tuning are unaffected by GABA blockage (Hechavarría and Kössl, 2014; Figure 5B). At the cortex, GABA blockage has a strong effect on the response amplitude. Altogether, the data indicate that PLS seen at the cortex level is inherited from subcortical structures, like the inferior colliculus or the thalamus. Future work, especially from the thalamus is required to fully understand the role of GABA and PLS on delay tuning.

Most cortical delay-tuned neurons are more sensitive to faint than to intense acoustic signals (Hechavarría and Kössl, 2014; Figure 5C). Non-monotonic intensity rate functions are less abundant in the inferior colliculus (Yang et al., 1992; Park and Pollak, 1993, 1994) than in the cortex (Suga and Manabe, 1982; Measor et al., 2018). In the pallid bat, non-monotonicity is more pronounced when using broadband FM sweeps than pure tones. This indicates that non-monotonicity is accentuated from sideband inhibition through dynamic interactions of different frequency inputs (Measor et al., 2018). The current view is that inhibitory inputs in the mammalian cortex are more broadly tuned than excitatory inputs (Wehr and Zador, 2003; Tan et al., 2004) and that there is an unbalanced recruitment between excitation and inhibition with increasing intensity (Wu et al., 2006). Experiments on thalamocortical brain slices from mice demonstrated that non-monotonicity can arise through local cortical inhibition (de la Rocha et al., 2008). While these findings, together with a formulated model, explain non-monotonicity in response to simple acoustic stimuli, they do not explain how non-monotonicity contributes to processing complex acoustic streams (de la Rocha et al., 2008), like echolocation sequences. By considering the presence of non-monotonicity, we adapted our current model of delay tuning (initial excitation + inhibition + post-inhibitory rebound) for the neural responses to the naturalistic echolocation sequences. Because of relatively low suppression, collicular neurons respond to almost each acoustic signal of our sequence. However, at the neuron’s best delay (6 ms in our example; Figure 5D) the post-inhibitory rebound from the call response coincides with the initial excitation of the echo response which results to a higher spike rate than non-optimal echo delays (blue arrowhead in Figure 5D). The very same echolocation sequence whose acoustic signals are well tracked by collicular neurons is totally differently encoded in the cortex. Here, massive suppression, in combination with non-monotonicity reduces the post-synaptic potentials induced by calls and echoes. Suppression effects last longer than in the midbrain and therefore may lead to subthreshold oscillations that reduce the neuron’s membrane potential. Although, subthreshold oscillations in cortical delay-tuned neurons await to be detected, similar oscillations have been detected in the inferior colliculus of bats and frogs (Covey et al., 1996; Feng, 2011). According to our model, relatively strong excitatory inputs are necessary to overcome cortical suppression and pass the spike threshold (Chen and Jen, 1994; Jen and Chen, 1998). This occurs when depolarizations induced by call and echo coincide (blue arrowhead in Figure 5E).

**FIGURE 6:**
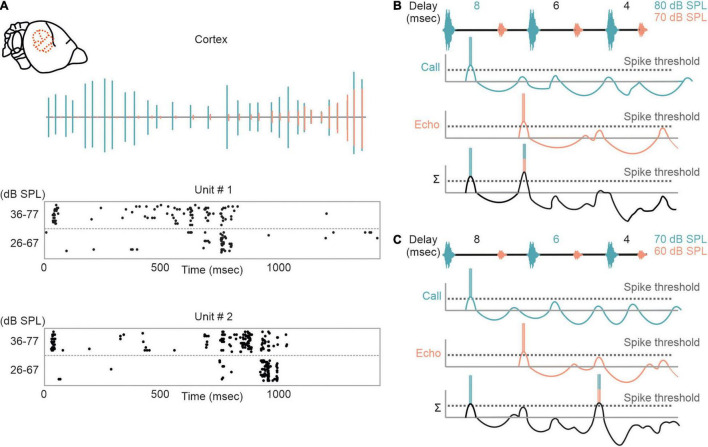
Intensity-dependent shifts in delay tuning in the auditory cortex. **(A)** Neural response of two cortical units to an echolocation sequence is shown as raster plots. (Top) Oscillogram of the sequence with calls and echoes depicted in green and orange, respectively. The sequence was presented at two different intensity ranges (36–77 and 26–67 dB SPL). Note, that the units respond earlier when the bat was stimulated with the high intensity range. **(B,C)** Proposed model explaining the intensity-dependent shift in delay tuning. When stimulated with an intense sequence (80–70 dB SPL), the non-monotonicity of the neuron elongates the inhibitory component and thus the rebound coincides with the initial excitation evoked by the first echo. Delay tuning gets shifted toward shorter delays (6 ms) when the bat gets stimulated with a fainter sequence (70–60 dB SPL). This results from a shortening of inhibition due to the non-monotonicity.

The influence of non-monotonicity is also reflected in cortical neurons that shift their delay tuning toward shorter delays with decreasing stimulus intensities (Figure 6A). According to the non-monotonicity, the duration of inhibition is prolonged with increasing intensity. Therefore, the inhibitory rebound (excitation II) delays with increasing intensity and thus the best delay is also shifted toward longer delays (Figures 6B,C).

Irrespective of the presented echo delay, many cortical neurons show an initial response to the echolocation sequence indicating for building up process of cortical suppression (Bartenstein et al., 2014; Beetz et al., 2016b). Initial responses are not essential for cortical suppression (Edamatsu and Suga, 1993; Beetz et al., 2016b) and in rodents it has been shown that even subthreshold depolarizations are followed by inhibition (Asari and Zador, 2009). Note that our model on processing naturalistic echolocation sequences is purely based on extracellular data obtained in anesthetized, passively listening bats of the species *C. perspicillata*. Our model does not consider the auditory thalamus whose influence on delay tuning is completely unknown in *C. perspicillata*. Future studies should consider performing neural recordings from multiple auditory stages, i.e., midbrain, thalamus, and cortex, at the same time in order to understand the organization of delay-tuning along the ascending auditory pathway. In addition, intracellular recordings monitoring the neuron’s membrane potential when the bats are stimulated with naturalistic echolocation sequences are essential.

## Neural Processing of Complex Naturalistic Scenes

Echolocation calls emitted in highly cluttered environments get reflected off multiple objects. This creates cascades of echoes following each call (Moss and Surlykke, 2010). Although bats may reduce the abundance of echo cascades by focusing their sonar beam to single objects (Surlykke et al., 2009a,b; Seibert et al., 2013; Fujioka et al., 2014), echo cascades cannot entirely be avoided (Linnenschmidt and Wiegrebe, 2016). Surprisingly, only a handful of studies tried to shed light onto how the bat’s brain processes spatial information from echo cascades in which the echoes were temporally non-overlapping (Beetz et al., 2016a,^2017^; Greiter and Firzlaff, 2017; Warnecke et al., 2018, 2021) [for neural results on temporally overlapping echoes that create spectral notches (see Sanderson and Simmons (2000, 2002), Allen et al. (2021))]. While neurons of the auditory midbrain parallelly process spatial information from multiple echoes (Beetz et al., 2017; Warnecke et al., 2018, 2021), the auditory cortex predominantly encodes the most immediate echo and subsequent echoes do not evoke a neural response (Beetz et al., 2016a; Greiter and Firzlaff, 2017). These data indicate that there must be a neural filter from the midbrain to the cortex. In anesthetized, passively listening bats, this filter selects the closest object. This is not a surprise when considering that the closest object represents the highest risk to collide with during flight. However, it is likely that attentional processes of the bat can adapt the filter so that more distant objects could be predominantly processed at cortical level, as it has been shown with behavioral experiments (Fujioka et al., 2016; Amichai and Yovel, 2017). Neural recordings from awake, echolocating bats are necessary to understand how this filter operates and how echo cascades are processed in the brain (Kothari et al., 2018).

Another major challenge accompanied by acoustic orientation is that bats live in a noisy environment enriched with communication and biosonar signals of conspecifics. This situation represents a cocktail party “nightmare” where bats are challenged to extract their own biosonar signals from a soup of noise. Although it has been shown that bats can discriminate their calls from interfering acoustic signals of conspecifics (Schnitzler et al., 1987; Brigham et al., 1989; Jones et al., 1991; Masters et al., 1991, 1995; Obrist, 1995; Amichai et al., 2015), call extraction becomes difficult with increasing noise level. To facilitate signal extraction, bats demonstrate a large repertoire of different behavioral adaptations. Some bats avoid flying in noisy environments (von Frenckell and Barclay, 1987; Beetz et al., 2019). However, due to the massive number of individuals sharing habitats, it is impossible to completely avoid acoustic interference. Additional adaptations are necessary to ensure signal extraction. Bats profit from their highly mobile outer ears. By adjusting the pinna position, they actively control their directional hearing (Gao et al., 2011; Wohlgemuth et al., 2016a). In addition, it has been shown that bats adjust different call parameters, including spectral, temporal, and energy level to ensure discriminability from conspecific signals (Simmons et al., 1975, 1978; Habersetzer, 1981; Miller and Degn, 1981; Suga et al., 1983; Roverud and Grinnell, 1985a; Obrist, 1995; Ibanez et al., 2004; Ratcliffe et al., 2004; Ulanovsky et al., 2004; Gillam et al., 2007; Gillam and McCracken, 2007; Petrites et al., 2009; Tressler and Smotherman, 2009; Hiryu et al., 2010; Hage et al., 2013; Takahashi et al., 2014; Amichai et al., 2015; Cvikel et al., 2015; Luo et al., 2015; Wheeler et al., 2016; Beetz et al., 2019, 2021).

**FIGURE 7:**
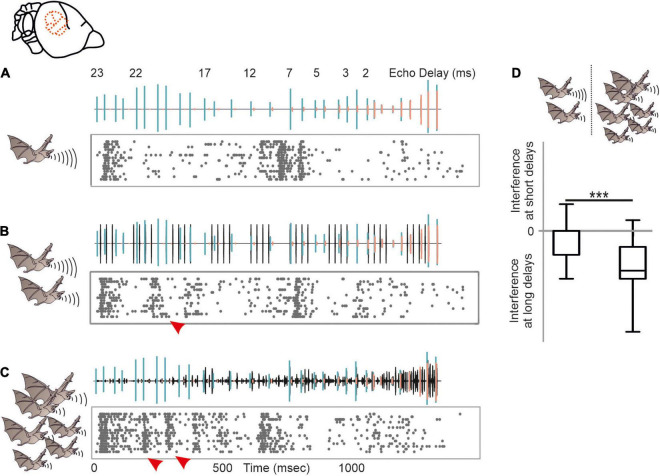
Acoustic interference arising from different naturalistic masker in the auditory cortex of *C. perspicillata*. **(A–C)** Neural activity of a cortical unit in response to the target sequence alone **(A)**, target sequence embedded in a moderate interferer (scenario when two bats echolocate) **(B)**, and target sequence embedded in strong interferer (scenario encountered in a bat colony) **(C)**. Oscillograms of the stimuli are shown above each raster plot. Respectively, call and echoes of the target stimulus are indicated in green and orange, while the interferer is shown in black. **(D)** Acoustic interference, shown as additional spikes in (*red arrowheads* in **B**,**C**) that were absent in **(A)**, preliminary occurs at the beginning of the sequence where long delays of the target sequence were dominant (Kruskal–Wallis test and Dunn’s multiple comparison *post hoc* test: ^***^p < 10^– 5^). Data from Beetz et al. (2018).

Despite of the large amount of reported behavioral strategies to overcome acoustic interference, we barely know how dramatic signal processing is affected by different naturalistic acoustic interferers at the neuronal level. We therefore tested the potential of acoustic interference of two different maskers on echolocation processing (Beetz et al., 2018). As a control, a target stimulus, represented by a naturalistic echolocation sequence was presented to an anesthetized bat (Figure 7A). The target sequence was embedded in two different maskers, a moderate and a strong masker. The moderate masker represented repetitive echolocation calls, a common acoustic scenario when two bats closely echolocate (Figure 7B). The strong masker represented an acoustic sequence recorded from a bat colony and contained echolocation and communication signals (Figure 7C). While neural processing of the target was mildly affected in the presence of the moderate masker, the strong masker substantially affected target processing (Beetz et al., 2018). Interestingly, acoustic interference, represented by additional spikes due to the presence of the masker were most abundant at the first half of the target sequence that exclusively contained long echo delays (*red arrowheads* in Figure 7). This means that call-echo pairs with long delays are more prone to acoustic interference than call-echo pairs with short delays. Our neural data fits very well to behavioral data on signal extraction in the presence of acoustic interferers in bats (Roverud and Grinnell, 1985b). By conditioning bats in a distance discrimination task and challenging them with timely controlled acoustic interferers, Roverud and Grinnell showed that delay tuning may be based on an integration time window. Each emitted echolocation call opens an integration time window in which the bat is highly sensitive to any subsequent acoustic signal (Roverud and Grinnell, 1985b; Neuweiler, 1990). Any acoustic signal following a call emission is automatically interpreted as a corresponding echo and closes the time window. If no acoustic signal follows, the time window gets automatically closed between 27 and 30 ms after call emission, depending on the bat species. The idea of an integration time window gets supported by our data (Beetz et al., 2018). Acoustic interferers only affect delay tuning, when they occur between call emission and echo arrival (Suga et al., 1983; Beetz et al., 2018). After echo arrival, the integration time window gets closed, and the neurons are not sensitive to any interferer. The existence of an integration time window receives support from our findings from the echo cascade processing in the cortex (Beetz et al., 2016a). Because the first echo automatically closes the integration time window, subsequent echoes are not processed, and cortical neurons process the first echo of an echo cascade. The aforementioned neural filter of the auditory cortex goes in line with the idea of an integration time window which seem to be non-existent in subcortical structures like the inferior colliculus (Beetz et al., 2017, 2018).

**FIGURE 8:**
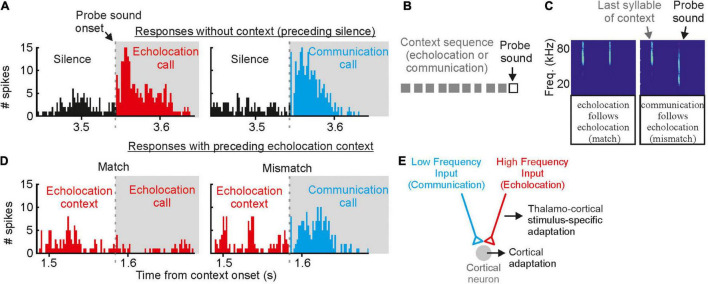
Contextual modulation of auditory responses in the auditory cortex of awake bats. **(A)** Example cortical unit that responds equally well to echolocation and communication sounds preceded by silence. **(B)** Schematic representation of the stimulation paradigm in which context (gray squares) precede a probe sound. **(C)** Spectrograms depicting the last syllable of context and the following probe sound. Two cases are shown: (1) match (echolocation follows echolocation) and (2) mismatch (communication follows echolocation). **(D)** Responses of the same cortical unit as in **(A)** to the echolocation and communication probes preceded by an echolocation sequence. Although the response to the probe was suppressed in both cases, the suppression was less severe in the mismatch. **(E)** Circuit that best explains the results obtained *in vivo* and modeling experiments. Data from Lopez-Jury et al. (2021).

While the moderate masker only contained high frequency echolocation signals, the strong masker contained both, high frequency echolocation signals and low to high frequency communication signals. We wondered whether the acoustic context, i.e., echolocation and communication may differently affect neural tuning to echolocation or communication signals. This is of special interest when considering that delay-tuned neurons of the cortex have multipeaked frequency tuning curves whose peaks match the peak frequencies of echolocation and communication signals (Hagemann et al., 2010, 2011; Hechavarría et al., 2016a,b, 2020). This raises the question whether echolocation and communication streams are processed in parallel by different subsets of neurons or whether the neurons are, depending on the current behavioral context, more sensitive to echolocation or communication signals? With neural recordings from the auditory cortex of awake *C. perspicillata*, we demonstrated that the neural sensitivity to the context, i.e., echolocation and communication, is strongly affected by the context of preceding sounds (Lopez-Jury et al., 2021). If non-selective neurons, i.e., neurons responding to both echolocation and communication calls, get primed by echolocation signals, they become selective for communication signals with a suppressed response to echolocation calls (Figure 8). When the very same neurons get primed with communication signals, then they become more sensitive to lagging echolocation than communication signals. This means that neural suppression is context dependent, and that the cortex is highly sensitive to novel sounds. These results are somehow counterintuitive because they imply that cortical neurons are weakly sensitive to echolocation signals when the bat echolocates, a situation when the neurons should be highly adapted to process echolocation signals. However, although, cortical suppression does reduce neural sensitivity it enhances neural selectivity by sharpening delay tuning (Beetz et al., 2016b). A sensitivity to novel stimuli is also known from stimulus specific adaptations (SSA) according to which neurons are highly sensitive to deviants (novel, unexpected) when they are adapted to standards (repetitive stimuli) (Calford and Semple, 1995; Ulanovsky et al., 2003; Carbajal and Malmierca, 2018). The study on bats evidently shows the behavioral relevance of context dependent neural adaptations (Lopez-Jury et al., 2021).

When comparing results obtained in bats that are often described as “auditory specialists” with data obtained in non-specialized mammals, it becomes evident that “auditory specialists” must cope with the same neurophysiological phenomena including SSA, forward suppression which may push the bat’s auditory system to its biological limits. Interestingly, both forward suppression and SSA do not necessarily represent a shortcoming of processing fast and repetitive stimuli but rather help the bat’s auditory system to selectively process behaviorally relevant stimuli.

## Neural Recordings From Awake and Echolocating Bats

**FIGURE 9:**
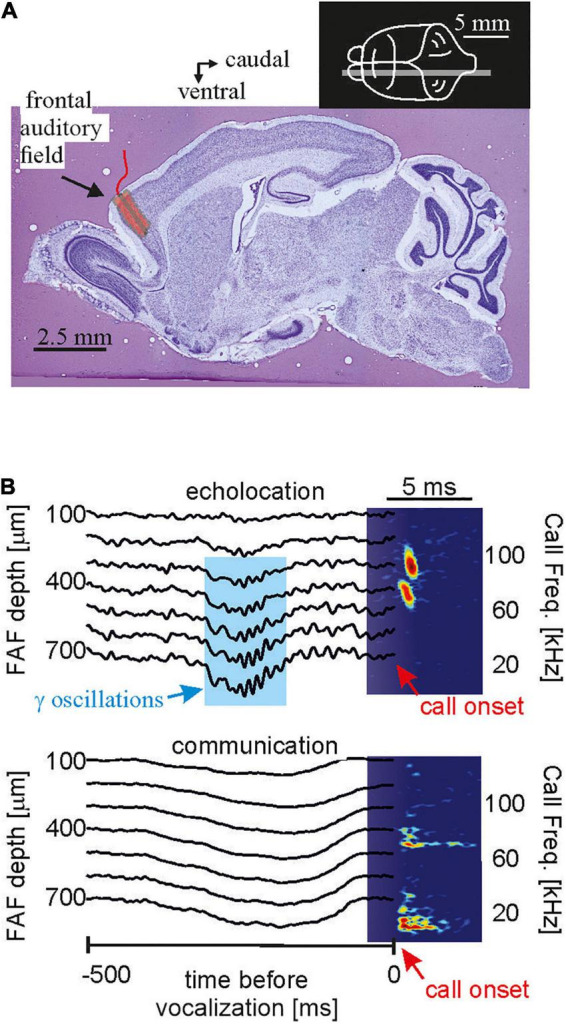
In the bat frontal cortex, gamma oscillations exclusively occur before echolocation but not communication emission. **(A)** Location of the frontal auditory field in a sagittal brain section of the bat *Carollia perspicillata.* **(B)** Neural activity across lamina of the frontal cortex before the production of one echolocation (*upper*) and one communication call (*lower*). Call spectrograms are given as colormaps. Data from Weineck et al. (2020).

One major goal of neuroscience is to obtain neural activity from animals while the animals show the behavior of interest. Most of the data reviewed here were obtained in lightly anesthetized passively listening bats and thus the validity of the current model of delay-tuning must be tested in actively vocalizing bats. Along the same line of arguments, it is unclear whether a similar cortical suppression in response to naturalistic echolocation sequences occurs in vocalizing animals. Studies on cortical coding of communication sequences have discovered neurons that can keep up with fast repetition rates when the bats were awake but non-vocalizing (syllable trackers, García-Rosales et al., 2018). Such neurons were not reported in anesthetized bats (Hechavarría et al., 2016b). Though communication and echolocation call coding are not necessarily similar, it would be important to assess how the auditory cortex of awake bats processes echolocation sequences. What might be even more important than the awake state might be the vocalizing state. Despite of recent technological advances, neural recordings from actively echolocating bats have rarely been conducted (Sinha and Moss, 2007; Kothari et al., 2018; Weineck et al., 2020; García-Rosales et al., 2022). However, it is indisputable that attentional effects occurring during active vocalization may completely alter neural processing of echolocation signals. Experiments in head-restrained vocalizing bats have shown strong gamma neural rhythms, usually linked to active processes such as attention, coupled to the production of echolocation calls in frontal cortices (Figure 9). Such gamma oscillations are less pronounced before the production of communication calls. The same study showed that frontal areas, which are likely involved in vocalization initiation, couple their activity with sensory-motor structures such as the striatum after echolocation (Weineck et al., 2020). In addition, information flows between frontal and auditory cortices reverses directionality after bats echolocate (García-Rosales et al., 2022) indicating that the results could be completely different in vocalizing compared to non-vocalizing bats.

Neural recordings from the auditory cortex and the inferior colliculus, brain regions of great interest when performing experiments in non-vocalizing bats are rare from vocalizing bats (Kawasaki et al., 1988; García-Rosales et al., 2022). Neural recordings in vocalizing bats that are stimulated with a replay of previously emitted echolocation signals are necessary to understand how vocalization and attentional processes affect neural coding. Irrespective of the comparability of data obtained in vocalizing (Kothari et al., 2018) and non-vocalizing bats (Beetz et al., 2016a,^2017^; Greiter and Firzlaff, 2017), it is noteworthy that under both recording conditions, neurons predominantly process the closest object when encountering echo information from multiple objects. These results show that conclusions drawn under artificial experimental settings, i.e., in passively listening bats, are worth to compare with experiments done under more naturalistic conditions. With the development of miniature recording devices (Marx, 2021), scientists route the technological requirements that are necessary to unravel neural mechanisms of naturalistic behavior. Not only recording devices, but also naturalistic contexts, like habitat, need to be reconstructed in order to understand neural processing of naturalistic behavior. Hereby, constructing elaborate tunnel mazes with a naturalistic representative size represents another big challenge to unravel the neural mechanisms of bat navigation behavior in future projects (Eliav et al., 2021).

## Conclusion

While enormous progress has been made over the last years in understanding how naturalistic echolocation sequences are processed in the bat brain, we are still far away of understanding how the brain controls echolocation behavior. To get a deeper understanding on the neural circuits and mechanisms of echolocation behavior, it is fundamental to perform studies under naturalistic stimulus contexts. Intracellular recordings from bats listening to naturalistic echolocation sequences may shed light on the neural mechanisms at the circuit level. At the same time, the development of electrodes that allow researchers to record from hundreds of neurons simultaneously (Hong and Lieber, 2019; Steinmetz et al., 2021; Gardner et al., 2022), gives an opportunity to unravel neural circuits at the neural population level. Of special interest, hereby, are simultaneous recordings from multiple brain regions along the ascending auditory pathway to understand how echolocation signals are processed in parallel. The importance of obtaining neural recordings from a population of neurons, rather than focusing on single neurons, becomes obvious when considering the spatial resolution encoded by delay-tuned neurons. While the bandwidth of delay-tuned neuron lies in the range of milliseconds (Feng et al., 1978; O’Neill and Suga, 1979; Hagemann et al., 2011), bats can discriminate delay differences of a few microseconds (Simmons, 1973, 1979). This discrepancy between neural and behavioral data may be resolved when considering a temporal coding strategy at a population level. According to a temporal code, which is often contrasted by a rate code, the spike time precision conveys information (Macías et al., 2020a). While (Beetz et al., 2016b; Luo et al., 2018) the spike time precision of a neuron varies hundreds of μs, the precision of extracellular field potentials (200–600 Hz), representing a summed response of a population of neurons varies by tens of μs (Luo et al., 2018). Thus, the behavioral data could theoretically be explained when considering data from a neuronal population that fires in synchrony, an effect that could not be characterized at single neuronal level.

Not only the stimulus context, but also the behavioral context must be carefully considered. Neurophysiological studies in vocalizing bats will be one major focus for future projects. However, at the same time, analyzing neural data from vocalizing bats represent another challenge. Each echolocation sequence is unique in its spectro-temporal properties and the animal’s attention which cannot necessarily be directly measured make the behavioral context highly variable, a scenario usually avoided by system neuroscientists that investigate neural processing in response to many invariant trials. Instead of trying to control physical properties of acoustic signals, scientists must focus to control the animal’s behavior and to average over multiple behavioral rather than stimulus trials. Current neurophysiological studies on freely flying bats mainly focus on performing neural recordings from relatively large fruit-bats while data from small, insectivorous bats are rare. The latter could be related to weight limitations, i.e., the maximum weight that small bats can carry. Therefore, ongoing advancement in developing small tracking and neural recordings devices, opens the possibility to investigate the neurobiology of echolocation behavior under more naturalistic conditions. By combining state-of-the-art recording approaches with naturalistic experimental conditions, scientists have enormous potential to lift the neuroethology of bat navigation to the next level in the upcoming years.

## Author Contributions

MJB wrote the first draft of the manuscript. JCH made contributions to the draft and funding acquisition. MJB and JCH prepared figures. Both authors contributed to the article and approved the submitted version.

## Conflict of Interest

The authors declare that the research was conducted in the absence of any commercial or financial relationships that could be construed as a potential conflict of interest.

## Publisher’s Note

All claims expressed in this article are solely those of the authors and do not necessarily represent those of their affiliated organizations, or those of the publisher, the editors and the reviewers. Any product that may be evaluated in this article, or claim that may be made by its manufacturer, is not guaranteed or endorsed by the publisher.
